# Clearance of Apoptotic Bodies, NETs, and Biofilm DNA: Implications for Autoimmunity

**DOI:** 10.3389/fimmu.2014.00365

**Published:** 2014-07-30

**Authors:** Marko Radic

**Affiliations:** ^1^Department of Microbiology, Immunology and Biochemistry, University of Tennessee Health Science Center, Memphis, TN, USA

**Keywords:** apoptosis, NETosis, clearance, autoimmunity, autoantibodies, lupus

## Introduction

**Figure 1:**
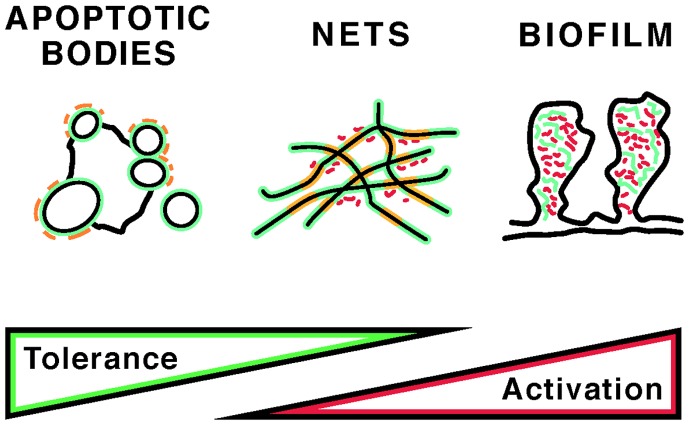
**Self and foreign antigens that may induce autoantibodies in autoimmunity**. The potential contribution of apoptotic bodies, NETs, and bacterial biofilms to immune tolerance versus stimulation is indicated. The distribution and content of self (green) and foreign (red) antigens is diagrammed. In apoptotic bodies, “foreign” structures may include post-translational modifications that are present only during late stages of apoptosis (orange). NETs, in addition to modified chromatin (orange), may also contain bacterial adjuvants, whereas biofilms may incorporate host DNA. Short red rods indicate bacteria in NETs and biofilms. For details, see text.

Apoptosis and NETosis, two important pathways of programed cell death, differ in their morphologic features and their effects on the immune system. In apoptosis, nuclear chromatin compacts as it is packaged into nuclear fragments and apoptotic blebs (1), and uptake of apoptotic cells by phagocytes generally suppresses the immune response (2). In NETosis, named after neutrophil extracellular traps (NETs), nuclear chromatin relaxes and forms a fibrous meshwork upon release from the cell (3). In general, NETosis is induced by infection, inflammation, or trauma and represents a mechanism of innate immune activation (4). Neutrophils, the most abundant type of white blood cells, migrate toward a stimulus in coordinated fashion, and NETs may synchronize such neutrophil swarms (5). Despite the structural and functional differences between apoptosis and NETosis, significant aspects of their clearance pathways likely overlap, as specific serum proteins participate in the recognition and uptake of remnants from either cell death pathway. *In vivo*, it is likely that both cell death pathways are concurrently present and that apoptotic bodies and NETs entangle (6). Yet, a third type of DNA may intertwine with DNA from apoptotic and NETotic cells, as certain bacteria and fungi release extracellular DNA that is used to construct biofilms (7). How apoptotic bodies, NETs, and biofilm DNA (Figure 1) are safely cleared is of great interest, because incomplete clearance leads to systemic inflammation and autoantibody production.

## Systemic Autoimmune Diseases and Autoantibodies to Nuclear Antigens

Molecular structures associated with dying cells are targets of autoantibodies in autoimmune diseases such as systemic lupus (SLE) (8), antiphospholipid syndrome (APS) (9), as well as other musculoskeletal/rheumatoid disorders (10). The resulting autoreactivities are idiosyncratic for each condition and thus are useful for clinical diagnosis. However, the antigens recognized by the autoantibodies are also involved in pathogenesis, as they accumulate at the sites of tissue damage and contribute to immune complex deposition (11). Tissue damage may worsen in the absence of serum nucleases such as DNAse I (12). Furthermore, the interactions between dying cells and the adaptive immune system strengthen over time, as somatic mutations and antigen selection optimize antibodies for improved binding (13). In SLE, antibodies to nuclear or plasma membrane antigens arise in the course of disease (14, 15). These antibodies avidly bind to apoptotic cells (16). Classical studies recognized that apoptotic cells are far better substrates for autoantibody binding than viable cells (17). However, monoclonal antibodies from mouse lupus models that bind to apoptotic blebs (16) also tightly bind to NETs released in response to bacterial pathogens (18). Our laboratory showed that NETotic cells provide suitable targets for autoantibodies from diverse human autoimmune disorders (19). Whether apoptotic or NETotic cell death, or both, provide antigens that induce autoantibody production is essential information for understanding the etiopathogenesis of autoimmune diseases (20).

## Apoptotic and NETotic Cell Death

Apoptosis is characterized by dramatic morphologic changes that are orchestrated by a family of specific proteases called caspases (21). The chromatin in the nucleus condenses tightly despite the fact that caspase-activated DNAse cleaves certain regions of genomic DNA to produce an oligonucleosome “ladder” (22). Curiously, the diameter (and thus the permeability) of nuclear pores transiently increases during this stage of apoptosis (23), and oligonucleosomes pass through the pores into the cytoplasm (16). The chromatin fragments associate with the outer nuclear envelope, the nucleus breaks up, and nuclear fragments migrate toward the cellular plasma membrane. These nuclear fragments form “blebs” at the cell surface, which are characteristic protrusions that give apoptotic cells their typical “grape cluster” appearance. Blebs display DNA, chromatin, and ribonucleoproteins at the cell surface (16, 24) such that these autoantigens become accessible to antibodies and pattern recognition receptors.

An alternative form of cell death was discovered by Brinkmann et al. (18). These authors reported that, upon exposure to bacteria, LPS, or PMA, neutrophils dissolve nuclear and cytoplasmic granule membranes, relax nuclear chromatin, associate the chromatin with granule components such as myeloperoxidase or elastase, and release the relaxed chromatin across the plasma membrane (4). The chromatin appears as disorganized fibers that spread widely to form an extracellular network. The authors named the fibers “NETs” because this chromatin could immobilize or “trap” bacteria. Mouse anti-chromatin antibodies were used to demonstrate that the NETs consisted of DNA and histones. These results immediately suggested that a tangle of bacteria and nuclear chromatin should be viewed as a “dangerous liaison” between lupus autoantigens and bacterial adjuvants that, by acting as a molecular complex, could trigger an adaptive immune response (25).

Follow-up studies revealed that NETs are not always an impediment to microbes. Proliferation assays identified certain species of bacteria that are resistant to any bactericidal effects of the released neutrophil chromatin (26), even though NETs organize bactericidal granule contents such as peroxidase and serine proteases (27), and even though histones also exhibit bactericidal activity (28). In fact, NET chromatin has found a novel use for certain bacteria that can incorporate NET chromatin into their extracellular matrix (29, 30). Such biofilms protect the microbes from physiological and pharmaceutical antibiotics and help to colonize various host tissues (7). DNA gives biofilms their structural integrity because nuclease treatment efficiently dissolves biofilms (31). The biofilms can also incorporate microbial DNA, as particular bacteria and fungi have mechanisms to release sections of genomic DNA for use in forming biofilms. Such DNA could be of particular significance in inducing anti-DNA responses because bacterial DNA has hypomethylated CpG motifs that directly stimulate toll-like receptors (32) and other DNA receptors (33) in B cells and other antigen- presenting cells.

## Evidence for Apoptosis and NETosis in the Induction of Autoimmunity

Evidence supporting apoptotic cells as the source of autoantigens that induce and promote the development of autoimmunity derives from a close inspection of autoantibody specificities. The observation that lupus serum IgG bind to apoptotic cells (17) initiated an active area of research. Because apoptotic cells externalize phosphatidylserine at the cell surface, binding of serum factors or lupus antibodies to phosphatidylserine could interfere with clearance in a way that would alter recognition of apoptotic cells and potentially induce disease. This view is consistent with genetic defects in cell clearance that in many instances recreate the full set of lupus manifestations (8).

Completion of the apoptotic program without adequate clearance may lead to the exposure of highly modified autoantigens (34). Autoantibodies to apoptotic cells may be induced by unique antigenic structures that are produced by enzymatic reactions in apoptotic cells. Granzyme B activation in apoptosis was identified as one possible mechanism whereby apoptosis generates novel self antigens that stimulate autoantibody binding (35). Importantly, characteristic post-translational modifications (PTM) of histones are induced during apoptosis. These include the acetylation of lysine 12 in the H2B core histone, a PTM that was shown to enhance the binding of lupus autoantibodies (36). However, lysine 12 acetylation also occurs in NETosis, and tri-acetylated histone H4, a specific target of the KM-2 murine lupus autoantibody, is more abundant in NETs from SLE patients than in controls (37). Therefore, antibody reactivity against any single histone PTM may not unambiguously establish which biological process supplies nuclear antigens in autoimmunity (38).

The generation of apoptotic cells during development and under conditions of rapid cell turnover, such as exist physiologically in primary lymphoid organs, suggests that apoptotic lymphocytes provide a steady supply of tolerogenic autoantigens (39). The idea that apoptosis provides self antigens that maintain tolerance is supported by immune suppression following injection of apoptotic cells (40). Immune suppression by apoptotic cells can also be recreated *in vitro* (41) and can be converted to immune activation by opsonization of apoptotic cells with antibodies (42). On balance, NETosis is a more likely alternative source of autoantigens that stimulate autoreactive B cells. This follows directly from the observation that, in autoimmunity, autoantibodies arise to various known NET components (43, 44). These include the proteases cathepsin G, proteinase 3, and elastase, as well as granule peptides, including LL37 and other defensins that have bactericidal properties.

Detailed analysis revealed that neutrophils from autoimmune patients are more prone to NETosis than controls and that NETosis is associated with particular autoantigen modifications (45, 46). Such autoantigen PTM may arise through reactive oxygen species liberated in NETosis or through enzymes that are activated during the progression of NETosis. Amino acids such as tryptophan and tyrosine are modified by oxidation or reactions with hypochlorous acid and peroxynitrite (47). NETosis also activates peptidylarginine deiminases (PADs), enzymes that convert arginine residues in proteins to citrulline residues. Our laboratory was first to link deimination (also known as “citrullination”) of nucleohistones to steps that are set in motion during NETosis (25). Importantly, we also showed that histone deimination is independent of caspase activity and that induction of apoptosis prevents PAD activation. Thus, deimination of histones clearly distinguishes NETosis from apoptosis.

In subsequent studies, we showed that citrullinated histones, including core and linker histones, are recognized in preference over non-modified histones by antibodies from patients with various autoimmune diseases, including SLE and Felty’s syndrome, a more severe form of rheumatoid arthritis (10). In confirming our results, others have shown that autoantibodies to deiminated histones are remarkably useful in the diagnosis of rheumatoid arthritis (48). In earlier studies, it was reported that citrullinated proteins are frequently targets of IgG antibodies from patients with arthritis (49), and antibodies to citrullinated antigens have been a focus of a growing number of research studies (50, 51). These observations represent a solid link between NETosis and the induction of disease-specific autoantibodies.

## Clearance Mechanisms

Clearance of apoptotic cells has been a focus of research for more than two decades (52), and a bewildering complexity of pathways has emerged (53). Different cell types participate in the uptake of apoptotic cells, the cells employ different combinations of receptors, and clearance may be enhanced or suppressed by various plasma proteins. Soluble plasma proteins that participate in apoptotic cell clearance include members of the pentraxin (54) and collectin families (55), the complement protein C1q (56), and milk fat globule epidermal growth factor 8 (MFG-E8) (57). An important “eat-me” signal is generated by the endoplasmic reticulum chaperone calreticulin. Apoptotic cells release calreticulin from the endoplasmic reticulum into the cytoplasm (58). The cytoplasmic calreticulin binds to phosphatidylserine in the inner leaflet of the plasma membrane from where it is externalized as the plasma membrane loses its asymmetry. At the cell surface, calreticulin combines with C1q and binds CD91 on the surface of the macrophage, leading to the phagocytosis of the apoptotic cell (59). Other receptors for uptake of apoptotic cells include SCARF1, a highly conserved receptor for C1q (60), and the integrin β_V_α_5_, a receptor for MFG-E8 (61). The importance of C1q, MFG-E8, and SCARF1 for tissue homeostasis is emphasized by the fact that mice deficient for any of these molecules show a reduced capacity for apoptotic cell clearance and exhibit a concomitant induction of autoantibodies (60, 62, 63). In SLE, altered levels of MFG-E8 in the serum and impaired C1q recognition of apoptotic cells correlate with the severity of disease manifestations (64, 65).

Additional receptors for the recognition and clearance of apoptotic cells are the Mer, Axl, and Tyro3 receptor tyrosine kinases (66). Mice deficient in any of these receptors manifest symptoms of autoimmune disease (67), and patients show altered serum levels of Mer family ligands GAS6 and protein S (68). Whereas Axl determines apoptotic cell clearance by dendritic cells (69), Mer is induced by C1q and serves to enhance apoptotic cell uptake by macrophage (70). It is important to note that several of these receptor–ligand systems are not specific for apoptotic cells but instead participate in the clearance of infectious microbes such as bacteria, fungi, and viruses (53). Possibly, some of these clearance pathways also serve to eliminate other cellular remnants.

Little is known about the clearance of NETotic cells, although a systematic analysis of the relevant mechanisms for NET clearance is urgently needed. Good starting points would be proteins and receptors that bind DNA or chromatin and that participate in the clearance of apoptotic cells. For example, several pentraxins (71) and collectins (55) bind to nucleic acids and chromatin, and calreticulin exhibits high affinity for chromatin and nucleosomes (72). It is likely that these proteins and receptors also bind NETs, although NETs are not efficiently recognized by the pentraxin C-reactive protein, or the complement protein C3b (73). In contrast, C1q binds NETs and activates the complement cascade (74, 75). The search for additional factors that regulate NET clearance is timely because NETosis has been linked to atherosclerosis (76), small vessel vasculitis (77), deep vein thrombosis (78), and various autoimmune conditions (79). Conversely, autoimmune diseases show an aberrant persistence of NETs, and NET clearance is impaired in APS (80), SLE (81), and gout (82). A better knowledge of NET clearance is expected to lead to new treatments for autoimmune diseases, as inhibitors of PAD4 show promise in various animal models of autoimmune disorders (83–86).

## Conflict of Interest Statement

The author declares that the research was conducted in the absence of any commercial or financial relationships that could be construed as a potential conflict of interest.
